# Role of Immunohistochemistry in the Diagnosis and Clinicopathological Stratification of Gliomas

**DOI:** 10.7759/cureus.105288

**Published:** 2026-03-16

**Authors:** Pragya Jaiswal, Mamatha K, Basavaraj Badadal

**Affiliations:** 1 Pathology, Shri BM Patil Medical College, BLDE (Deemed to be University), Vijayapura, IND; 2 Neurosurgery, Shri BM Patil Medical College, BLDE (Deemed to be University), Vijayapura, IND

**Keywords:** atrx, gfap, glioma, idh1 (r132h) immunopositivity, immunohistochemistry, ki-67

## Abstract

Introduction

Gliomas are common primary brain tumors. Immunohistochemical components, such as isocitrate dehydrogenase (IDH) mutations, GFAP (glial fibrillary acidic protein), ATRX (alpha-thalassemia/mental retardation, X-linked), and Ki-67, play a pivotal role in glioma diagnostic classification and risk stratification in prior literature. This study aimed to evaluate the association of IDH1 (R132H) IHC as a surrogate marker within the World Health Organization (WHO) integrated framework and to correlate the expression of IDH1 (R132H) with clinicopathological parameters in gliomas.

Methods

A hospital-based cross-sectional study was conducted on 30 histologically confirmed glioma cases, with an age range from 1 to 70 years. Clinicopathological parameters such as age of the patient, gender, tumor location, histological type, and World Health Organization tumor grade were recorded. Immunohistochemistry for IDH1 (R132H), Ki-67, GFAP, and ATRX was performed. Associations were assessed using Fisher's exact test (2×2 tables) and Fisher-Freeman-Halton exact test (r×c tables), with p < 0.05 considered significant.

Results

IDH1 (R132H) immunopositivity was seen in 19 cases (63.3%). ATRX retention was observed in 63.3% of cases, GFAP positivity in 93.3%, and a high Ki-67 index in 50% of cases. IDH1 (R132H) IHC status showed a statistically significant association with age, histological type, and WHO grade. No statistically significant association was observed with gender, ATRX, GFAP, or Ki-67 expression.

Conclusion

IDH1 (R132H) immunohistochemical expression showed a significant association with established clinicopathological indicators, including patient age, histological type, and WHO tumor grade. In this hospital-based cross-sectional study, IDH1 (R132H) IHC-negative status was more frequently observed in higher-grade tumors. These findings support the use of immunohistochemistry as an accessible adjunct in glioma diagnosis and clinicopathological stratification; outcome-based prognostic significance requires longitudinal follow-up.

## Introduction

Gliomas represent a major proportion of primary malignant brain tumors and remain among the most clinically challenging central nervous system tumors due to their aggressive activity and low long-term survival in high-grade tumors [1]. The worldwide incidence rate of glioma is 4.28 per 100,000 population [2]. Males (3.9 per 100,000 population) typically have greater incidence rates than females (3.0 per 100,000 population) [3].

According to Global Burden of Disease 2021 estimates, malignancies of the central nervous system have a significant negative impact on health, with an age-standardized disability-adjusted life year (DALY) rate of almost 113 per 100,000 people. The global age-standardized mortality rate for central nervous system (CNS) malignancies was reported to be 3.06 per 100,000 population [2]. Gliomas are classified and graded using histomorphology criteria and assigned WHO grade 1 through grade 4. WHO grade 3 and 4 tumors, which are frequently combined to form high-grade gliomas, account for around 75% of all gliomas [4]. Among adult-type diffuse gliomas, astrocytic tumors represent a major subgroup [1].

The CNS World Health Organization (WHO) 2021 classification provides an integrated approach in which molecular alterations complement histology for tumor typing and grading. Key molecular features used in adult diffuse glioma classification include isocitrate dehydrogenase (IDH) mutation status, 1p/19q codeletion status, alpha-thalassemia/mental retardation syndrome X-linked (ATRX) alteration, epidermal growth factor receptor (EGFR) amplification, and telomerase reverse transcriptase (TERT) promoter mutation [5,6].

Since the WHO 2016 update and reinforced in WHO 2021, isocitrate dehydrogenase (IDH) mutation study has become a central component of the integrated diagnosis of diffuse gliomas and is also widely recognized as a marker with prognostic implications in prior literature. IDH1 (R132H) immunopositivity is frequently encountered in lower-grade diffuse gliomas and is reported in a substantial proportion of WHO grade 2 and grade 3 tumors. IDH has three different isoforms: IDH1 exists in the cytoplasm and peroxisomes, whereas IDH2 and IDH3 are prevalent in the mitochondrial matrix. Accordingly, assessment of IDH status, often using IDH1 (R132H) immunohistochemistry in routine practice, assists in integrated classification and clinicopathological stratification of gliomas. IDH mutations most commonly involve IDH1 and IDH2; in routine histopathology practice, IDH1 (R132H) immunohistochemistry is commonly used as a practical surrogate for integrated diagnosis [7]. In prior literature, glioma subgroups stratified by IDH status have shown distinct clinical and genomic profiles; in routine practice, IDH1 (R132H) immunohistochemistry serves as a practical surrogate marker, but IHC-negative cases require molecular testing for definitive classification. Glioblastoma multiforme (CNS WHO grade 4) is associated with poor long-term survival despite multimodal therapy [8].

One of the common alterations in IDH-mutant astrocytoma is ATRX inactivation, and ATRX immunohistochemistry is useful in distinguishing astrocytoma (ATRX loss is common) from oligodendroglioma (ATRX retention with 1p/19q codeletion) within the WHO 2021 framework [9]. ATRX retention may also occur in IDH1 (R132H) IHC-negative glioblastoma multiforme, so ATRX must be interpreted with morphology and integrated workup.

Glial fibrillary acidic protein (GFAP) expression is considered highly specific for astrocytic differentiation, which makes it one of the most common immunohistochemistry indicators in diagnostic neuropathology and is expressed in a broad spectrum of astrocytic tumors, whereas it is generally absent or only focally expressed in oligodendroglial and neuronal tumors. In low-grade astrocytoma, GFAP expression is generally strong and diffuse, indicating preserved astrocytic differentiation. In contrast, high-grade diffuse gliomas, including glioblastoma multiforme, IDH1 (R132H) IHC-negative, frequently exhibit heterogeneous, patchy, or even focal staining due to tumor dedifferentiation and significant cellular heterogeneity [10].

Ki-67 is a commonly used immunohistochemical marker of cellular proliferative activity in tumors. In diffuse gliomas, higher Ki-67 labeling indices are generally more frequent in higher-grade lesions and can support assessment of proliferative activity. However, Ki-67 is not an absolute predictor of tumor behavior and should be interpreted in conjunction with histomorphology and integrated diagnostic classification. Lower-grade gliomas typically demonstrate lower Ki-67 indices compared to high-grade gliomas, although variability may be seen across cases and histological variants [11]. While high-grade diffuse gliomas, glioblastoma multiforme exhibits significantly elevated levels, which are associated with rapid proliferation, increased cellular atypia, and a worse clinical outcome, lower-grade gliomas usually show a low Ki-67 index. In oligodendrogliomas and astrocytoma, elevated Ki-67 is reported in several studies and shows frequent association with shorter survival and a higher risk of recurrence [12].

In many centers, especially where comprehensive molecular testing is limited, immunohistochemistry provides an accessible approach to support integrated diagnosis. This study aimed to evaluate the association between IDH1 immunohistochemical expression and clinicopathological parameters, and to examine its relationship with ATRX, GFAP, and Ki-67 in histologically confirmed gliomas.

An integrated approach combining histology with key immunohistochemical markers improves diagnostic reproducibility and supports prognostic assessment in gliomas [13].

Aim of the study

This study aims to evaluate the diagnostic utility of immunohistochemistry in gliomas and its association with clinicopathological parameters and established prognostic indicators, including WHO grade.

## Materials and methods

This was a hospital-based cross-sectional study conducted on 30 patients, with an age range of 1-70 years, who were diagnosed with glioma between March 2024 and October 2025 in the Histopathology Section of Shri BM Patil Medical College Hospital and Research Centre, BLDE (Deemed to be University), Vijayapura, India. Institutional ethical clearance was obtained for this study.

**Figure 1:**
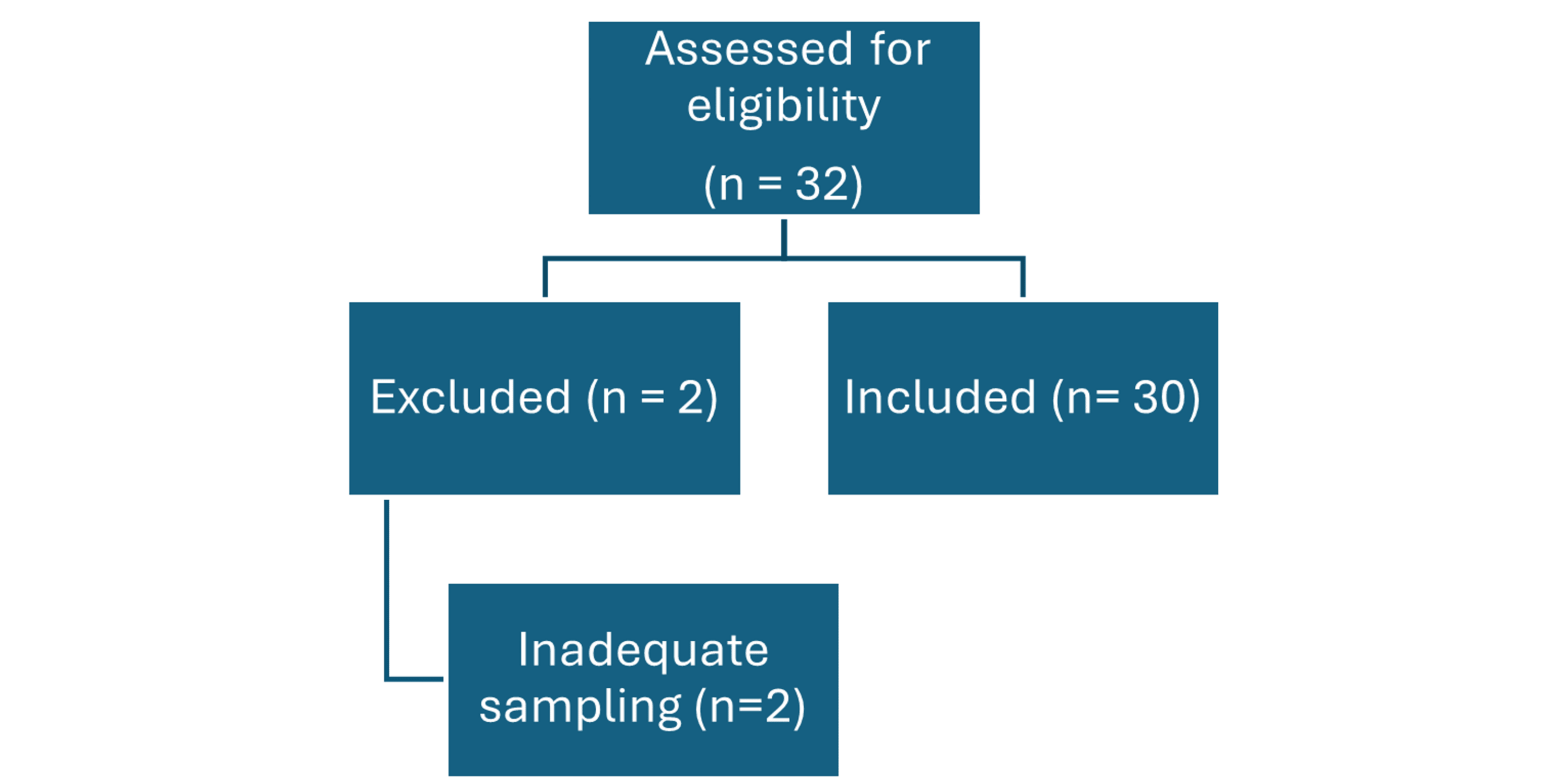
Flowchart depicting the inclusion and exclusion criteria

Histologically confirmed cases of gliomas were included, and tissues with inadequate sampling were excluded (Figure 1). The cohort included both pediatric and adult patients (age range: 1-70 years). Cases were classified using histomorphology and the available immunohistochemical panel (IDH1 (R132H), ATRX, GFAP, Ki-67) in accordance with routine diagnostic practice at our center. Comprehensive molecular testing required for age-specific integrated classification (including pediatric-type glioma categorization) was not available; therefore, pediatric cases were analyzed within the same histopathology/IHC-based framework and interpreted cautiously.

The tissue specimens were fixed in 10% buffered formalin and processed routinely. Four-micrometer sections were cut from paraffin-embedded tissue blocks. One section was stained with hematoxylin and eosin (H&E) for histopathological diagnosis.

Another four sections from the same block were mounted on poly-L-lysine-coated slides from paraffin-embedded tissue blocks, which were subjected to IDH1 (performed using clone H09 (R132H) from Dianova, dilution 1:20, on the Ventana Benchmark platform, with heat-induced antigen retrieval (CC1 buffer), OptiView detection system, and known IDH1-mutant glioma as positive control), ATRX (performed using clone D5 from Sigma-Aldrich, dilution 1:100, on the Ventana Benchmark platform, with heat-induced antigen retrieval (CC1 buffer), OptiView detection system, and retained nuclear staining in endothelial cells as internal positive control), GFAP (performed using clone GA5 from Dako/Agilent, dilution 1:500, on the Ventana Benchmark platform, with heat-induced antigen retrieval (CC1 buffer), OptiView detection system, and normal brain tissue as positive control), and Ki-67 (performed using clone MIB-1 from Dako/Agilent, dilution 1:100, on the Ventana Benchmark platform, with heat-induced antigen retrieval (CC1 buffer), OptiView detection system, and tonsil or lymph node as positive control) immunohistochemical staining. Each IHC run included appropriate positive and negative controls, and staining adequacy was confirmed prior to interpretation.

IDH1 expression was scored based on cytoplasmic staining intensity and proportion of positive tumor cells. ATRX expression was evaluated based on nuclear immunoreactivity. Ki-67 labeling index was calculated as the percentage of positively stained nuclei among at least 1000 tumor cells. GFAP expression was recorded as positive or negative based on cytoplasmic staining in tumor cells.

IDH1 immunohistochemical expression was correlated with clinicopathological variables such as patient age, gender, tumor location, tumor histological type, WHO tumor grade, and other immunohistochemical markers.

Interpretation criteria for IHC markers

For diagnostic categorization, IDH1 (R132H) was interpreted primarily as positive (tumor cell cytoplasmic staining) or negative; the semiquantitative score was used only for standardized documentation and subgroup analysis.

Data were entered into Microsoft Excel and analyzed using IBM SPSS Statistics for Windows, Version 20 (Released 2011; IBM Corp., Armonk, New York). Categorical variables were summarized as frequencies and percentages. The association between IDH1 (R132H) IHC status and clinicopathological variables was evaluated using exact tests because of the small sample size and sparse contingency tables. Fisher's exact test was used for 2×2 tables, and the Fisher-Freeman-Halton exact test was used for r×c tables. All tests were two-tailed, and p < 0.05 was considered statistically significant.

ATRX Marker

ATRX was interpreted as retained when tumor nuclei showed nuclear staining comparable to internal control cells (endothelial cells/neurons/reactive glia). Loss was defined as the complete absence of nuclear staining in tumor cells with preserved staining in internal control cells.

GFAP Marker

GFAP was considered positive when tumor cells demonstrated cytoplasmic immunoreactivity; staining was recorded as diffuse versus focal or patchy where applicable.

Ki-67 Marker

Ki-67 labeling index (LI) was calculated as the percentage of positively stained tumor nuclei among the total tumor nuclei counted, using the formula (number of Ki-67-positive tumor nuclei / total tumor nuclei counted) × 100. Hotspot areas were selected by scanning the section to identify regions showing the highest density of Ki-67-positive nuclei, and counting was then performed in these areas. A minimum of 1000 tumor cells were counted per case. Scoring was performed manually by a single pathologist; interobserver concordance analysis was not performed. Ki-67 was categorized using a predefined semiquantitative scoring system as follows: Score 0 (<1%), Score 1 (1-5%), Score 2 (6-10%), Score 3 (11-20%), and Score 4 (>20%).

## Results

**Figure 2:**
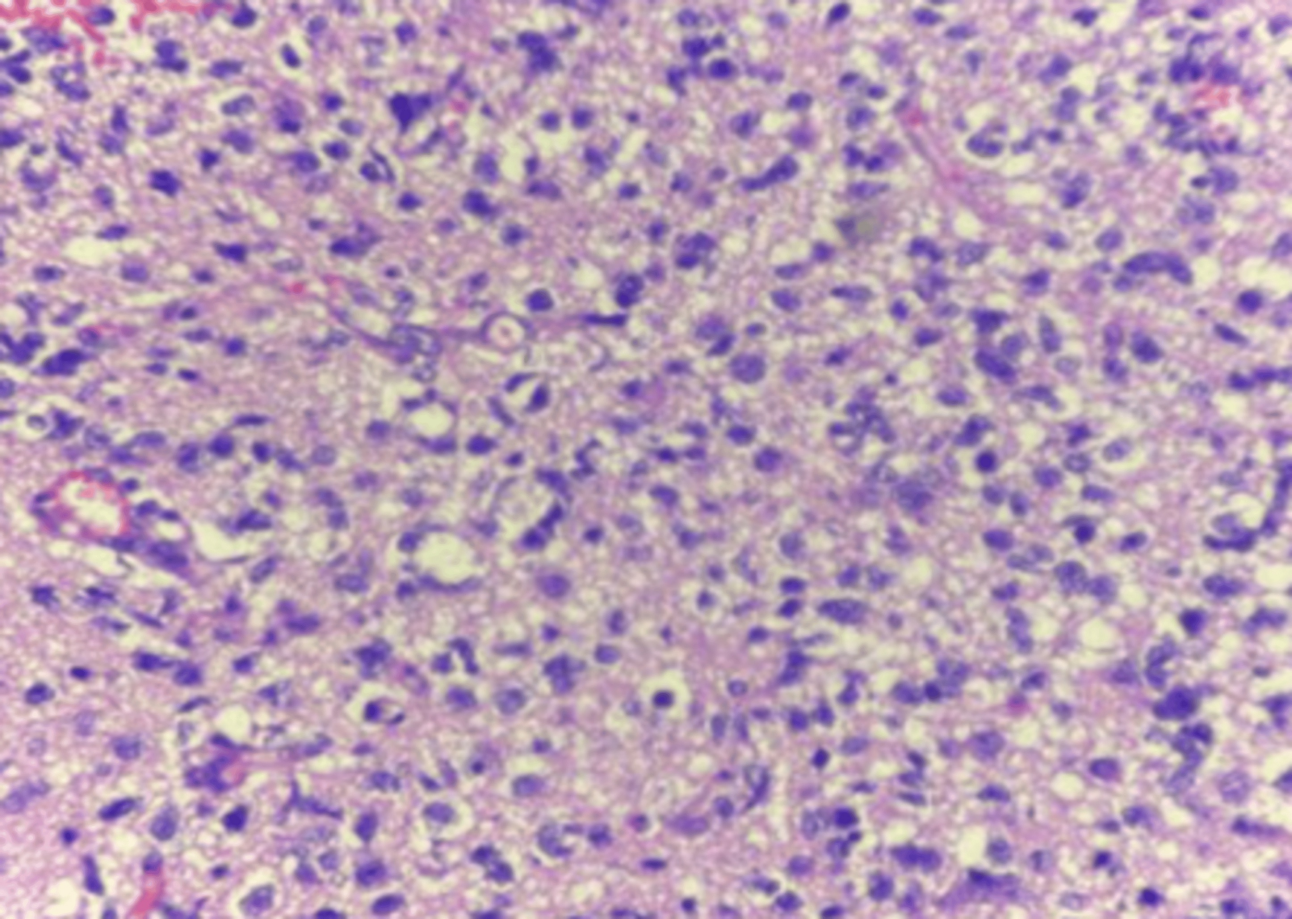
Photomicrograph of astrocytoma: histologic grade 3 (H&E) (400x)

**Figure 3:**
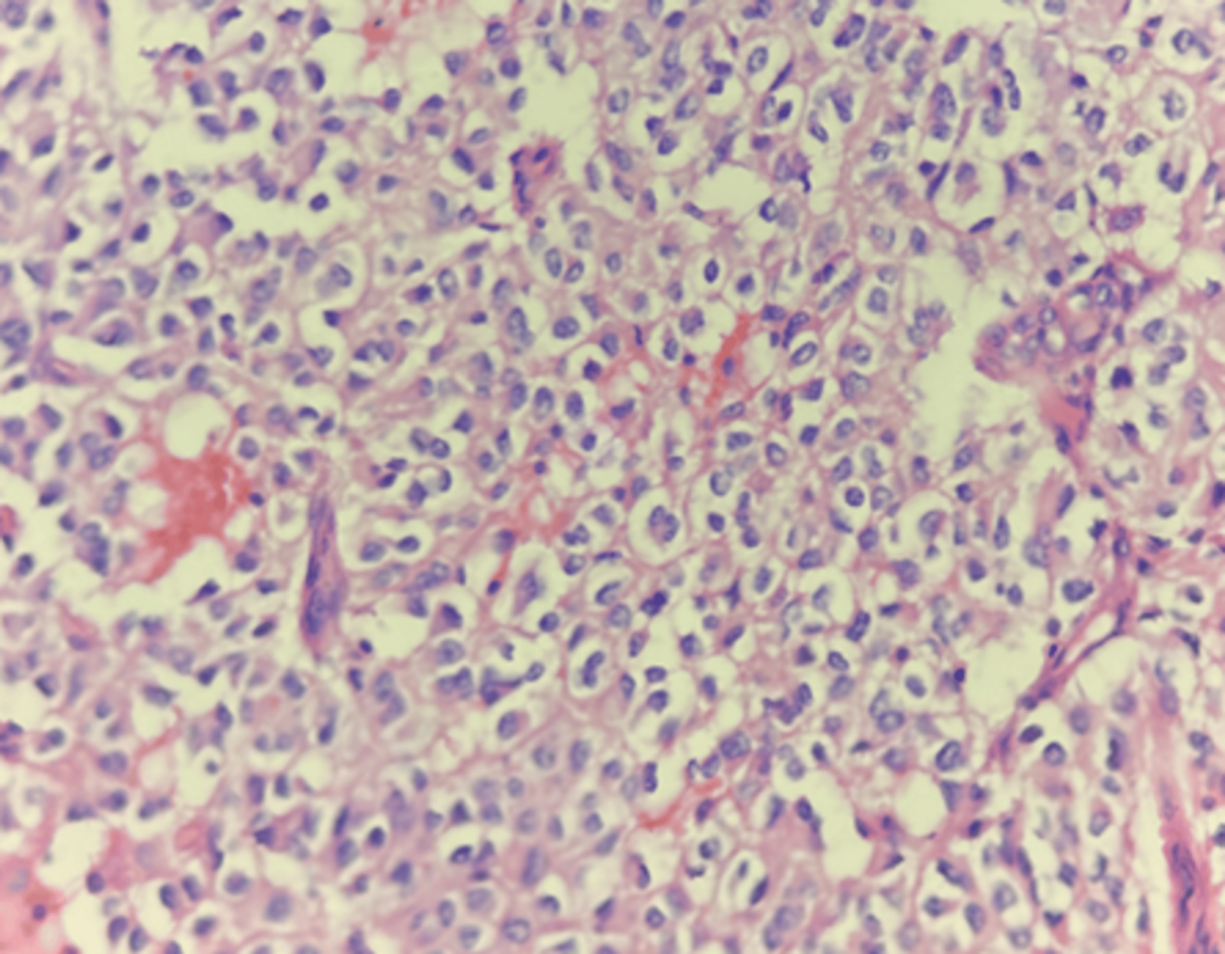
Photomicrograph of oligodendroglioma: histologic grade 3 (H&E) (400x)

**Figure 4:**
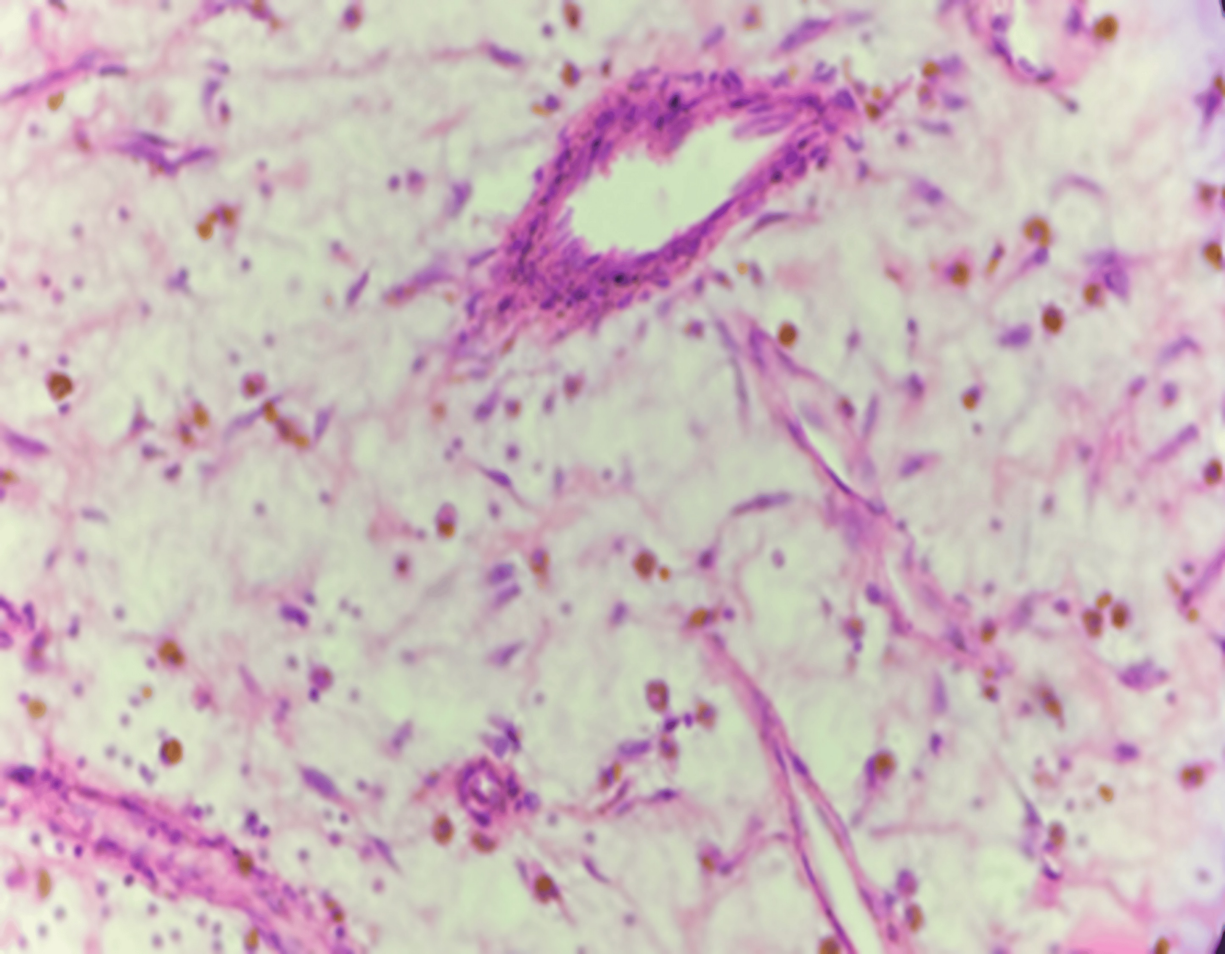
Photomicrograph of glioblastoma multiforme: histologic grade 4 (H&E) (400x)

The present study included 30 patients diagnosed with Glioma on histopathology (Figures 2-4).

**Figure 5:**
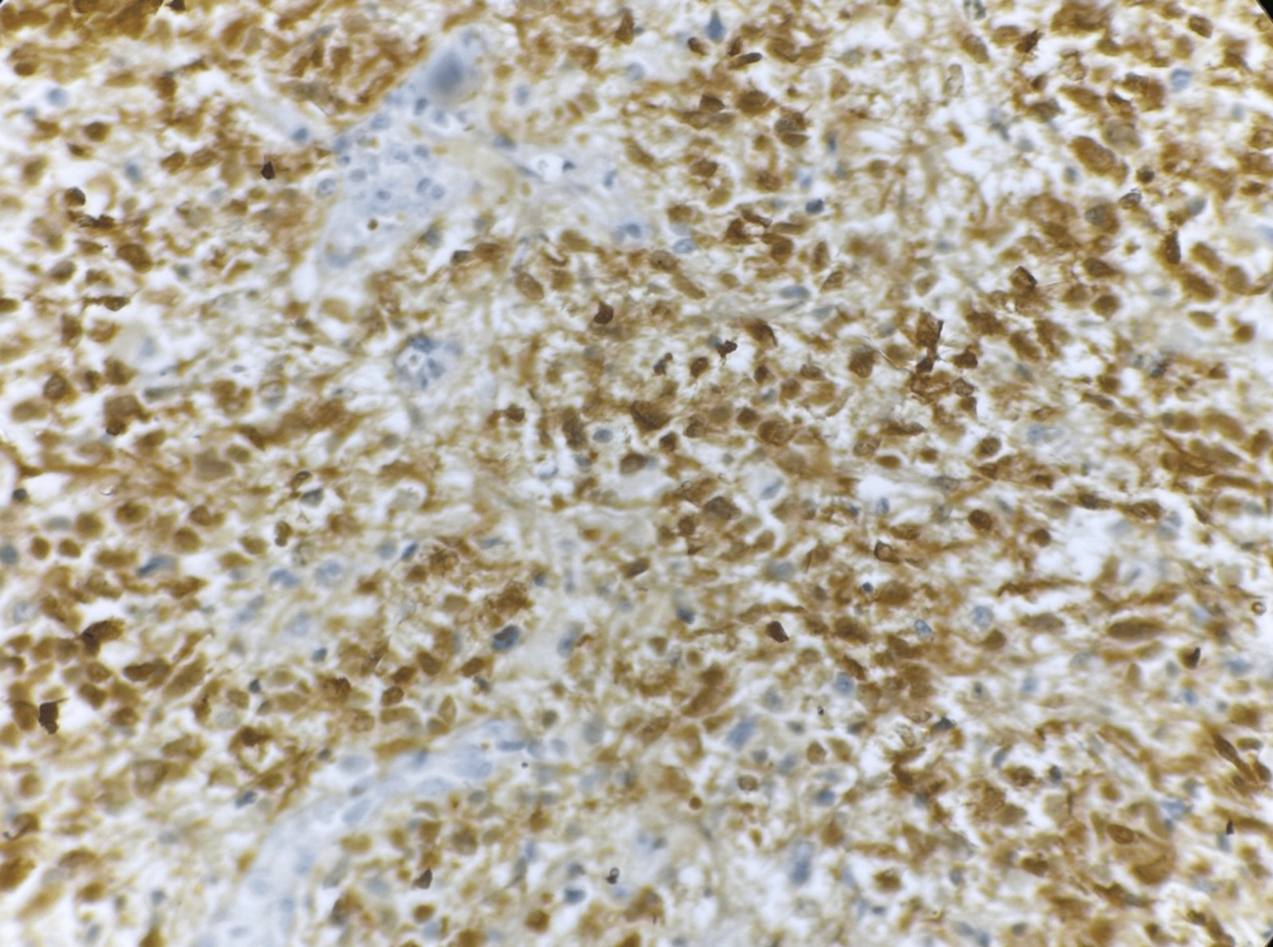
Photomicrograph of immunohistochemistry (IHC) marker isocitrate dehydrogenase 1 (IDH1) showing cytoplasmic positivity

**Figure 6:**
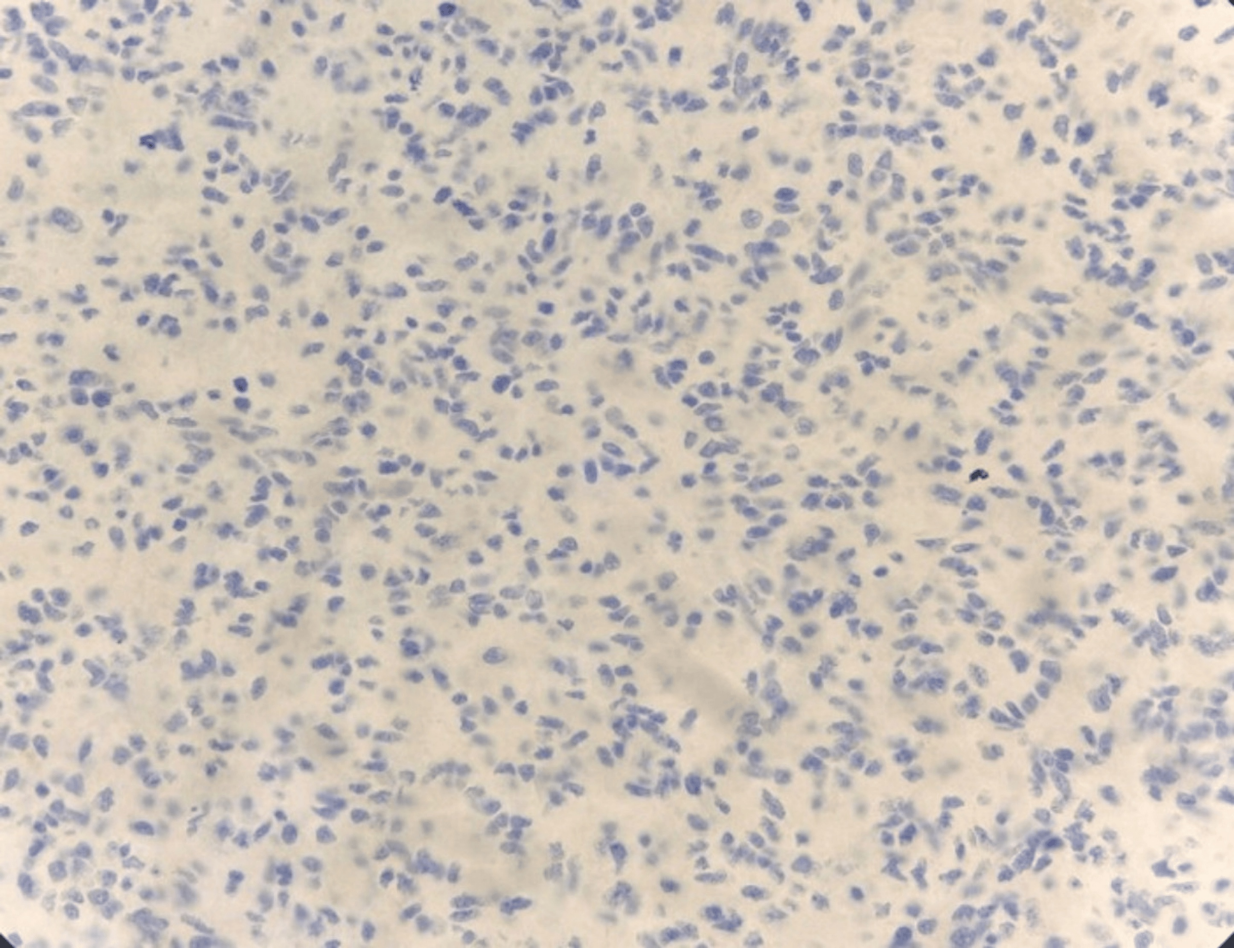
Photomicrograph of immunohistochemistry (IHC) marker isocitrate dehydrogenase 1 (IDH1) showing negative staining for IDH1

The quantity and intensity of IDH1 expression were assessed in tumor cells. All IDH1-positive cases demonstrated cytoplasmic staining. IDH1 (R132H) immunopositivity was observed in 19 cases (63.3%), as shown in Figure 5, and these were classified as IDH1 (R132H) immunopositive. Eleven cases were IDH1 (R132H) IHC-negative, as shown in Figure 6, and were classified as IDH1 (R132H) IHC-negative.

**Figure 7:**
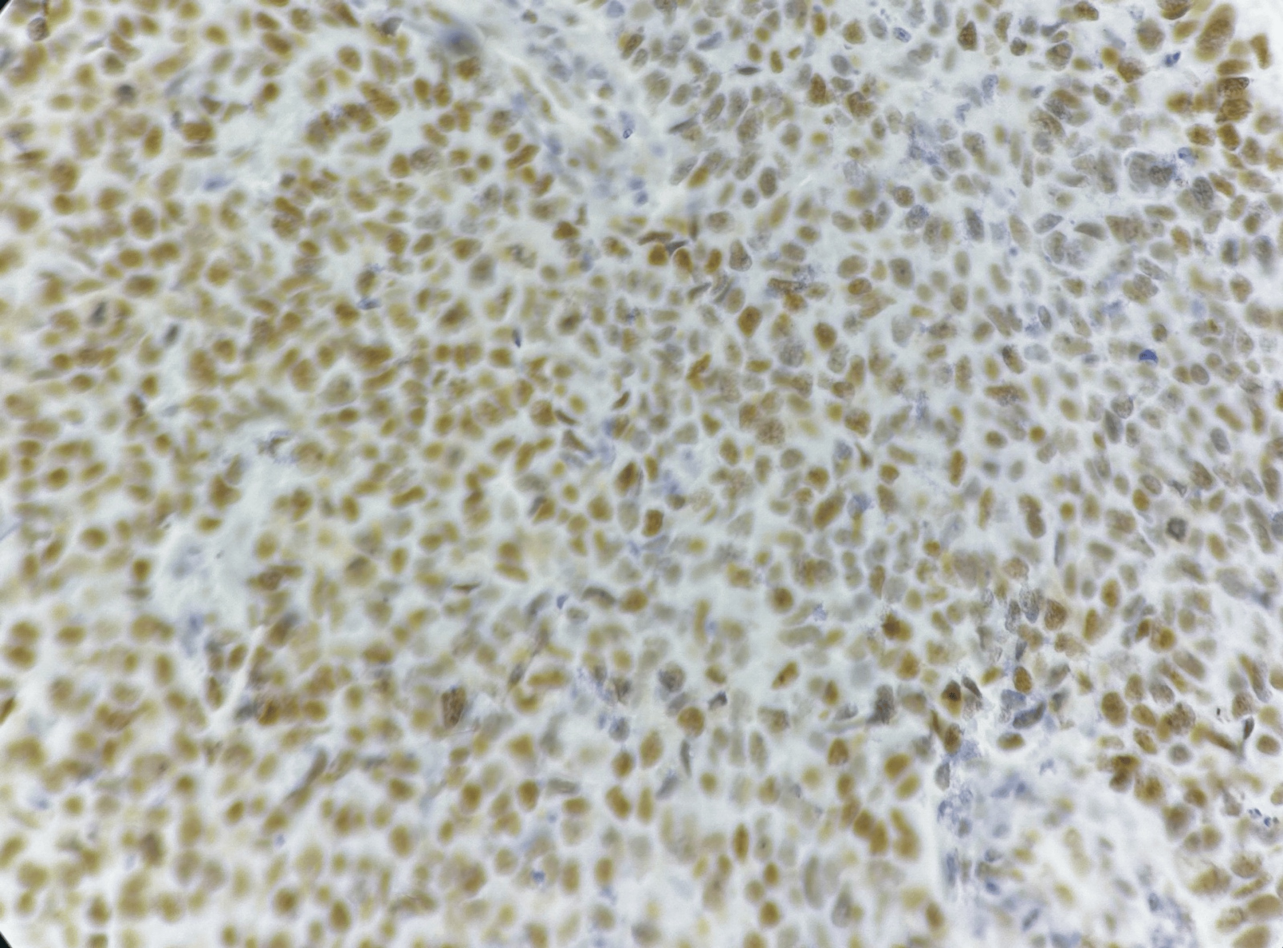
Photomicrograph of immunohistochemistry (IHC) marker alpha-thalassemia/mental retardation syndrome X-linked (ATRX) showing retention in internal control

**Figure 8:**
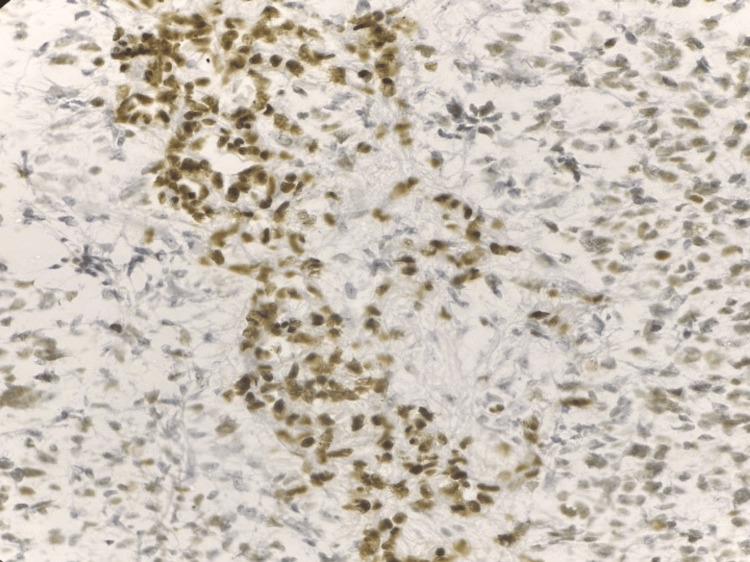
Photomicrograph of immunohistochemistry (IHC) marker alpha-thalassemia/mental retardation syndrome X-linked (ATRX) showing nuclear positivity

ATRX expression was evaluated in tumor cells. ATRX-positive cases showed well-defined nuclear staining in neoplastic cells, with retained staining in internal control cells (Figures 7, 8). ATRX expression was retained in 19 cases (63.3%).

**Figure 9:**
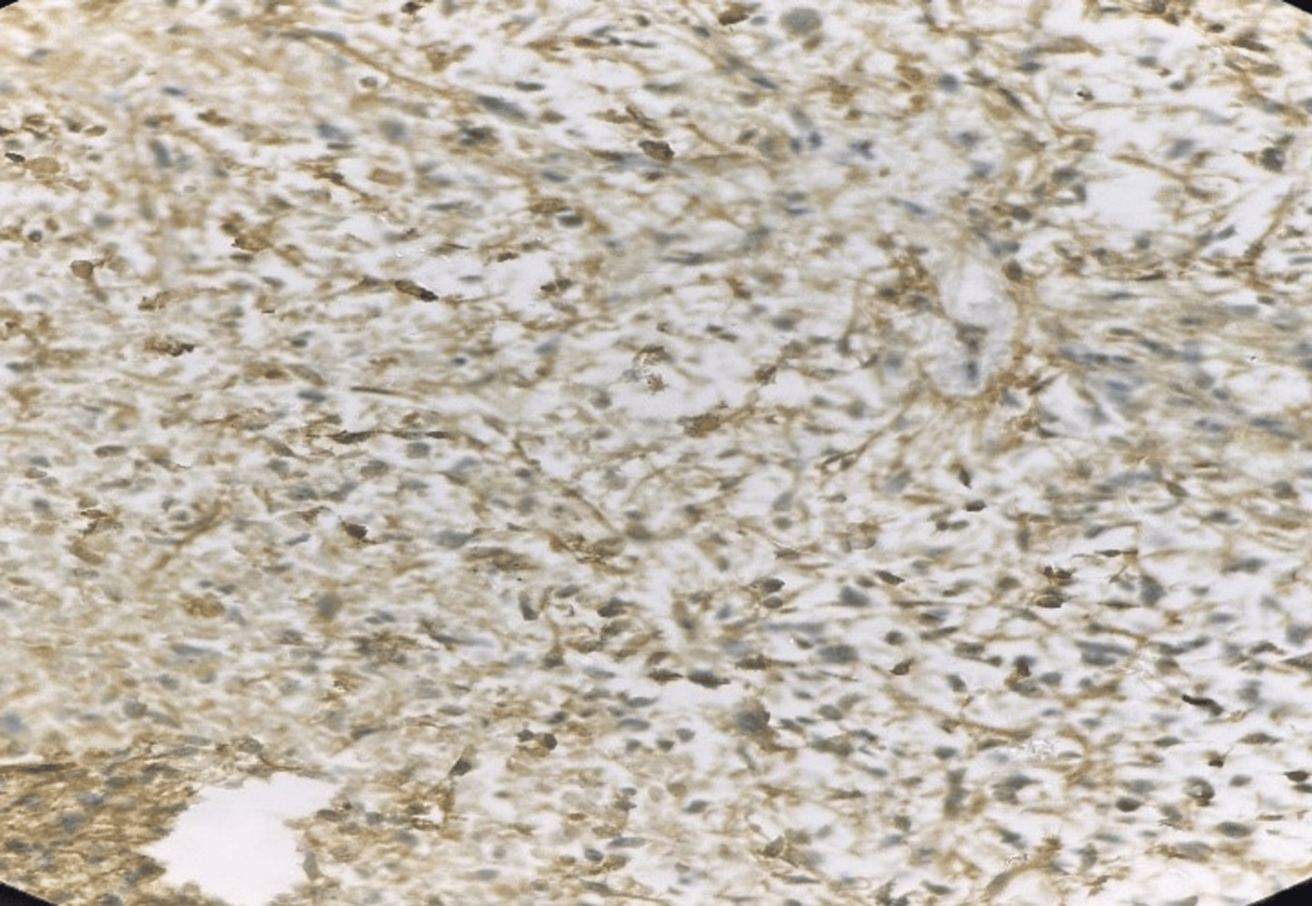
Photomicrograph of immunohistochemistry (IHC) marker glial fibrillary acidic protein (GFAP) showing cytoplasmic positivity

GFAP immunohistochemistry was performed in all cases. All GFAP-positive cases demonstrated strong, diffuse cytoplasmic staining in tumor cells (Figure 9). GFAP immunopositivity was observed in 28 cases (93.3%), while two cases were GFAP-negative; interpretation was made in conjunction with histomorphology and the overall IHC profile.

**Figure 10:**
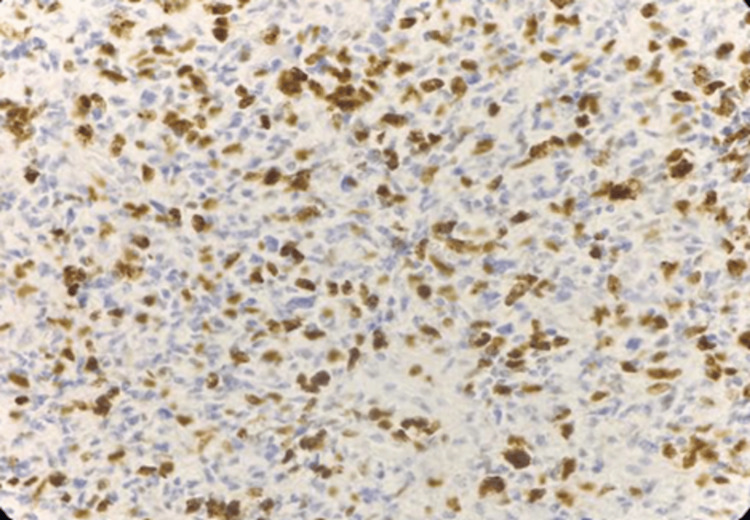
Photomicrograph of Ki-67 immunohistochemistry showing nuclear positivity in tumor cells (representative case from the high Ki-67 expression group)

Ki-67 immunohistochemistry was performed in all cases. In positive cases, tumor cells exhibited nuclear staining (Figure 10). Fifteen cases (50.0%) showed high Ki-67 expression, four cases (13.3%) showed moderate expression, and 11 cases (36.7%) showed low expression. For analysis, Ki-67 expression was grouped as low (Score 0-1; <1% to 5%), moderate (Score 2; 6-10%), and high (Score 3-4; ≥11%), as defined in the Methods.

The age of patients with glioma ranged from 1 to 70 years, with a mean age of 43.2 years. Of the 30 cases, seven (23.3%) were in the <30-year age group, and all seven (100.0%) showed IDH1 (R132H) IHC positivity. In the 30-50-year age group (n = 9, 30.0%), seven cases (77.8%) were IDH1 (R132H) IHC-positive. Among patients aged >50 years (n = 14, 46.7%), IDH1 (R132H) IHC positivity was seen in five cases (35.7%), while the remaining nine cases (64.3%) were IHC-negative. A statistically significant association was observed between IDH1 (R132H) IHC status and age group (Fisher-Freeman-Halton exact test, p = 0.010) (Table 1).

Of the 30 cases, 17 (56.7%) were male and 13 (43.3%) were female, with a male-to-female ratio of 1.3:1. IDH1 (R132H) IHC positivity was observed in 13 of 17 male patients (76.5%) and 6 of 13 female patients (46.2%). However, no statistically significant association was found between IDH1 (R132H) IHC status and gender (Fisher's exact test, p = 0.132) (Table 1).

**Table 1:** Association of IDH1 (R132H) IHC status with clinicopathological parameters Ki-67 LI was assessed in hotspot areas (the highest density of positive nuclei) by manual counting of ≥1000 tumor cells. Ki-67 scores were defined as: Score 0 (<1%), Score 1 (1–5%), Score 2 (6–10%), Score 3 (11–20%), and Score 4 (>20%). For analysis, Ki-67 expression was grouped as low (Score 0–1), moderate (Score 2), and high (Score 3–4). Exact tests were used due to sparse cells/zero counts: Fisher’s exact test for 2×2 tables and Fisher–Freeman–Halton exact test for r×c tables. All tests were two-tailed; p<0.05 was considered significant. IDH categories represent IDH1 (R132H) IHC-positive vs IHC-negative and are not equivalent to molecular IDH-mutant vs IDH-wildtype without sequencing. IDH1: isocitrate dehydrogenase 1, IHC: immunohistochemistry, WHO: World Health Organization, ATRX: alpha-thalassemia/mental retardation syndrome X-linked, GFAP: glial fibrillary acidic protein, Ki-67: marker of cellular proliferation, LI: labelling index.

Parameter	Category	IDH1 (R132H) IHC-positive (n=19)	IDH1 (R132H) IHC-negative (n=11)	Test used	p-value
Age	<30 years	7 (100.0%)	0 (0.0%)	Fisher–Freeman–Halton exact	0.010
	30–50 years	7 (77.8%)	2 (22.2%)		
	>50 years	5 (35.7%)	9 (64.3%)		
Gender	Male	13 (76.5%)	4 (23.5%)	Fisher’s exact	0.132
	Female	6 (46.2%)	7 (53.8%)		
Histological type	Astrocytoma/Oligodendroglioma	19 (100.0%)	0 (0.0%)	Fisher’s exact	<0.001
	Glioblastoma	0 (0.0%)	11 (100.0%)		
WHO grade	Grade 2	10 (100.0%)	0 (0.0%)	Fisher–Freeman–Halton exact	<0.001
	Grade 3	3 (100.0%)	0 (0.0%)		
	Grade 4	6 (35.3%)	11 (64.7%)		
ATRX expression	Positive	13 (68.4%)	6 (31.6%)	Fisher’s exact	0.696
	Negative	6 (54.5%)	5 (45.5%)		
GFAP expression	Positive	18 (64.3%)	10 (35.7%)	Fisher’s exact	1.000
	Negative	1 (50.0%)	1 (50.0%)		
Ki-67 expression	Low	9 (81.8%)	2 (18.2%)	Fisher–Freeman–Halton exact	0.342
	Moderate	2 (50.0%)	2 (50.0%)		
	High	8 (53.3%)	7 (46.7%)		

With respect to tumor location, IDH1 (R132H) IHC positivity was observed in 13 of 20 frontal lobe tumors (65.0%), three of four temporal lobe tumors (75.0%), two of five parietal lobe tumors (40.0%), and the single occipital lobe tumor (100.0%). Tumor location was recorded as a descriptive clinicoradiological variable, and no inferential statistical analysis was performed for this parameter.

Histologically, 19 of 30 cases (63.3%) were classified as astrocytoma/oligodendroglioma, and all 19 cases (100.0%) showed IDH1 (R132H) IHC positivity. The remaining 11 cases (36.7%) were diagnosed as glioblastoma, and all 11 cases (100.0%) were IDH1 (R132H) IHC-negative. IDH1 (R132H) IHC status showed a statistically significant association with histological type (Fisher's exact test, p < 0.001) (Table 1).

With regard to WHO grade, all grade 2 tumors (10/10, 100.0%) and all grade 3 tumors (3/3, 100.0%) were IDH1 (R132H) IHC-positive. Grade 4 tumors (n = 17, 56.7%) included six IDH1 (R132H) IHC-positive cases (35.3%) and 11 IHC-negative cases (64.7%). Overall, grade 4 tumors constituted the largest subgroup and showed a predominance of IDH1 (R132H) IHC negativity, whereas lower-grade tumors (grades 2 and 3) uniformly demonstrated IHC positivity. A statistically significant association was observed between IDH1 (R132H) IHC status and WHO tumor grade (Fisher-Freeman-Halton exact test, p < 0.001) (Table 1).

The cohort included three pediatric patients (<18 years; ages three, 10, and 12 years). All three pediatric cases were histologically classified as astrocytoma and were IDH1 (R132H) IHC-positive. To assess whether inclusion of pediatric cases influenced the observed associations, a sensitivity analysis was performed in the adult-only subgroup (≥18 years, n = 27). The direction and significance of the major findings remained unchanged: IDH1 (R132H) IHC status remained significantly associated with age group, histological type, and WHO grade, while no significant associations were observed with gender, ATRX, GFAP, or Ki-67 expression.

No statistically significant association was observed between IDH1 (R132H) IHC status and gender, ATRX expression, GFAP expression, or Ki-67 expression category.

## Discussion

Gliomas remain one of the most challenging central nervous system tumors due to their biological heterogeneity and variable clinical outcomes. Although recent advances in molecular diagnostics have improved tumor classification and clinically relevant stratification, the full spectrum and functional significance of genetic and epigenetic alterations in glioma genesis remain to be delineated. Despite advances in neurosurgical techniques and adjuvant therapies, treatment outcomes remain limited, particularly in high-grade gliomas such as glioblastoma. Hence, continued research is vital to improve early detection, treatment, and survival [13].

Histopathological overlap between astrocytoma and oligodendroglioma can lead to diagnostic uncertainty, particularly in the absence of molecular testing (1p/19q codeletion, IDH sequencing) in low-resource settings. Hence, more studies can validate IHC-based surrogate markers as cost-effective tools in such settings [14].

In the present study, we evaluated the association between IDH1 immunohistochemical expression and clinicopathological parameters, along with its relationship to ATRX, GFAP, and Ki67 in gliomas.

The mean age in this study was 43.2 years. The majority of the cases showing IDH1 (R132H) immunopositivity were below 50 years of age, whereas the majority of IDH1 (R132H) IHC-negative cases were above 50 years of age.

Our findings demonstrated a statistically significant association between IDH1 expression and patient age, with tumors showing IDH1 (R132H) immunopositivity occurring more frequently in younger patients. This observation is consistent with previously reported age-related differences across glioma subgroups in the WHO 2021 framework [1]. However, in the present study, IDH1 (R132H) IHC-negative cases were not interpreted as molecularly confirmed IDH-wildtype tumors in the absence of sequencing [1].

In the present study, the male-to-female ratio was 1.3:1. The majority of the male patients demonstrated IDH1 (R132H) immunopositivity. No statistically significant association was observed between IDH1 expression and patient gender, suggesting that IDH1 (R132H) immunopositivity status is independent of sex distribution in this cohort.

The frontal lobe was the most commonly involved tumor site (20/30 cases), and the majority of frontal lobe tumors showed IDH1 (R132H) IHC positivity. Tumor location was analyzed as a descriptive clinicoradiological variable, and no inferential statistical analysis was performed for this parameter.

The histological types of gliomas included in our study were astrocytoma, oligodendroglioma, and glioblastoma. Most cases were astrocytoma/oligodendroglioma (19/30, 63.3%), and all of these cases showed IDH1 (R132H) IHC positivity. All 11 glioblastoma cases were IDH1 (R132H) IHC-negative. IDH1 (R132H) IHC status showed a statistically significant association with histological type (p < 0.001), demonstrating a distribution pattern consistent with established clinicopathological classification. This is in line with Sharma et al. [15], who also reported a statistically significant association between histological category and IDH1 expression (p < 0.05).

Grade 4 lesions constituted the largest subgroup in the present study (17/30, 56.7%), which is comparable to the predominance of grade 4 tumors reported by Meel et al. [14], Priambada et al. [7], Sharma et al. [15], and Fatima et al. [16]. In our cohort, all IDH1 (R132H) IHC-negative cases belonged to WHO grade 4, whereas all grade 2 (10/10) and grade 3 (3/3) tumors were IDH1 (R132H) IHC-positive. Accordingly, a statistically significant association was observed between IDH1 (R132H) IHC status and WHO tumor grade (p < 0.001). These findings indicate that IDH1 (R132H) IHC-negative status was predominantly associated with higher-grade disease in this cohort. However, as IDH2 and noncanonical IDH1 mutations were not assessed by sequencing, IDH1 (R132H) IHC-negative status should not be equated with molecular IDH-wildtype.

In the present study, ATRX expression was retained in 19 cases (63.3%). Among IDH1 (R132H) IHC-positive cases, ATRX retention was seen in 13/19 cases (68.4%), while among IDH1 (R132H) IHC-negative cases, ATRX retention was observed in 6/11 cases (54.5%). ATRX was interpreted using internal controls and integrated morphologic context. Similar trends have been reported in prior studies, including Priambada et al. [7] and Meel et al. [14]. However, no statistically significant association was observed between ATRX expression and IDH1 (R132H) IHC status in our cohort (p = 0.696), which may reflect the limited sample size.

GFAP expression was observed in 28/30 cases (93.3%) in the present study. Among IDH1 (R132H) IHC-positive cases, GFAP positivity was seen in 18/19 cases (94.7%), while among IDH1 (R132H) IHC-negative cases, GFAP positivity was observed in 10/11 cases (90.9%). GFAP expression did not show a statistically significant association with IDH1 (R132H) IHC status (p = 1.000). In our study, GFAP was used as a supportive marker of glial differentiation, and isolated GFAP negativity was interpreted in conjunction with histomorphology and the overall IHC profile.

In our study, 15/30 cases (50.0%) showed high Ki-67 expression. Among cases with high Ki-67 expression, 8/15 (53.3%) were IDH1 (R132H) IHC-positive and 7/15 (46.7%) were IHC-negative. When analyzed by IDH1 (R132H) IHC status, high Ki-67 expression was observed in 8/19 IHC-positive cases (42.1%) and 7/11 IHC-negative cases (63.6%). Although a higher proportion of IDH1 (R132H) IHC-negative cases showed high Ki-67 expression, the association between Ki-67 expression category and IDH1 (R132H) IHC status was not statistically significant in this cohort (p = 0.342). This suggests that proliferative index and IDH1 (R132H) IHC status may represent distinct clinicopathological dimensions in this dataset. Overall, our findings should be interpreted as clinicopathological associations within a cross-sectional cohort rather than as outcome-based prognostic effects.

Strength of the study

The study provides a comprehensive immunohistochemical evaluation of gliomas and demonstrates significant associations between IDH1 (R132H) IHC status, patient age, histological type, and WHO grade. These findings contribute clinically relevant clinicopathological and epidemiological data and support the utility of IDH1 (R132H) immunohistochemistry as an accessible adjunct for glioma classification and clinicopathological stratification, particularly in resource-limited settings.

Limitations of the study

This study has several limitations. First, it was a hospital-based cross-sectional study, and longitudinal clinical outcomes (such as progression-free survival, overall survival, recurrence, and treatment response) were not assessed; therefore, direct prognostic conclusions cannot be drawn. Second, IDH sequencing was not performed, and IDH2 as well as noncanonical IDH1 mutations were not assessed; accordingly, IDH1 (R132H) IHC-negative cases cannot be equated with molecular IDH-wildtype tumors. Third, the cohort included both pediatric and adult patients (age range: 1-70 years), but comprehensive molecular profiling required for integrated age-specific classification, including pediatric-type glioma categorization, was not available. Therefore, pediatric cases were classified and analyzed using histomorphology and IHC findings, which may limit direct alignment with WHO integrated molecular categories.

## Conclusions

Immunohistochemistry remains a practical and valuable adjunct in the diagnostic evaluation of gliomas, particularly in resource-limited settings. In the present hospital-based cross-sectional study, IDH1 (R132H) IHC status showed a significant association with established clinicopathological indicators, including histological type and WHO grade, with IDH1 (R132H) IHC negativity more frequently observed in grade 4 tumors. These findings support the role of IHC in diagnostic classification and clinicopathological stratification. However, as longitudinal clinical outcomes were not assessed, no direct conclusions regarding prognosis or survival can be drawn from this study. Further studies with molecular confirmation and follow-up outcome data are required to establish prognostic significance.
